# A Proteomic Approach of *Bradyrhizobium/Aeschynomene* Root and Stem Symbioses Reveals the Importance of the *fixA* Locus for Symbiosis

**DOI:** 10.3390/ijms15033660

**Published:** 2014-02-28

**Authors:** Nathanael Delmotte, Samuel Mondy, Benoit Alunni, Joel Fardoux, Clémence Chaintreuil, Julia A. Vorholt, Eric Giraud, Benjamin Gourion

**Affiliations:** 1 Institute of Microbiology, Eidgenössische Technische Hochschule (ETH) Zurich, Vladimir-Prelog-Weg 4, 8093 Zurich, Switzerland; E-Mails: nathanael.delmotte@gmail.com (N.D.); vorholt@micro.biol.ethz.ch (J.A.V.); 2 Institut des Sciences du Végétal, CNRS, 91198 Gif sur Yvette, France; E-Mails: samuel.mondy@isv.cnrs-gif.fr (S.M.); alunni@isv.cnrs-gif.fr (B.A.); 3 Département de Biologie, Université Paris Sud, 91400 Orsay, France; 4 Laboratoire des Symbioses Tropicales et Méditerranéennes, IRD, UMR IRD/SupAgro/INRA/UM2/CIRAD, F-34398 Montpellier, France; E-Mails: joel.fardoux@ird.fr (J.F.); clemence.chaintreuil@ird.fr (C.C.)

**Keywords:** proteome, photosynthetic *Bradyrhizobium*, symbiotic nitrogen fixation, stem nodulation, *fix*A

## Abstract

Rhizobia are soil bacteria that are able to form symbiosis with plant hosts of the legume family. These associations result in the formation of organs, called nodules in which bacteria fix atmospheric nitrogen to the benefit of the plant. Most of our knowledge on the metabolism and the physiology of the bacteria during symbiosis derives from studying roots nodules of terrestrial plants. Here we used a proteomics approach to investigate the bacterial physiology of photosynthetic *Bradyrhizobium* sp. ORS278 during the symbiotic process with the semi aquatical plant *Aeschynomene indica* that forms root and stem nodules. We analyzed the proteomes of bacteria extracted from each type of nodule. First, we analyzed the bacteroid proteome at two different time points and found only minor variation between the bacterial proteomes of 2-week- and 3-week-old nodules. High conservation of the bacteroid proteome was also found when comparing stem nodules and root nodules. Among the stem nodule specific proteins were those related to the phototrophic ability of *Bradyrhizobium* sp. ORS278. Furthermore, we compared our data with those obtained during an extensive genetic screen previously published. The symbiotic role of four candidate genes which corresponding proteins were found massively produced in the nodules but not identified during this screening was examined. Mutant analysis suggested that in addition to the EtfAB system, the *fixA* locus is required for symbiotic efficiency.

## 1. Introduction

Rhizobia are soil bacteria that are able to establish symbiotic interactions with plants of the legume family. These interactions lead to the development of facultative organs called nodules that house bacteria. In the nodules, bacteria are living as intracellularly separated from plant cytoplasm by the peribacteroidal membrane. Once in the plant cell cytoplasm, intracellular rhizobia are referred as “bacteroids”. Together with the peribacteroidal membrane, bacteroids form the symbiosome. Bacteroids live under microoxic conditions and their metabolism undergoes drastic modifications, so does their morphology during the symbiosis with some legume species [1–3]. They produce nitrogenase and fix atmospheric nitrogen to generate ammonium which is transferred to the plant allowing its growth in soils where nitrogen is scarce. In return, plants provide fixed carbon to the bacteria in the form of dicarboxilic acids which are actively oxidized through the tricarboxilic acid cycle to produce ATP required for nitrogenase activity [1,4].

In most legume species, nodules are formed at the roots, albeit some tropical legumes, such as *Sesbania rostrata* [5] or species of the genus *Aeschynomene* [6] also develop stem nodules. Amongst the studies conducted on stem nodulation, a genetic screen on *Azorhizobium caulinodans* has been performed in order to identify rhizobial factors required for stem nodules maturation and maintenance [7]. Nevertheless, essentially all of the knowledge related to rhizobial physiology during the symbiotic process was derived from studies focused on root nodules. These studies were mainly conducted with bacteria interacting with few legume models or crop plants, including the *Bradyrhizobium japonicum–*soybean symbiosis and the *Sinorhizobium meliloti–Medicago truncatula* symbiosis. *Bradyrhizobium* sp. ORS278 establishes a symbiosis with plants of the genus *Aeschynomene* and is able to trigger nodule formation on roots as well as stems of its host. Moreover, the Alphaproteobacterium possesses three more atypical traits amongst rhizobia: its photosynthetic ability which is required for optimal stem symbiosis [8], its ability to fix nitrogen under free living condition [9] and the absence of the *nod* genes previously considered as universal amongst rhizobia [10].

These traits make *Bradyrhizobium* sp. ORS278 an interesting organism to investigate its physiology during the symbiotic process for which proteomics is a promising approach to uncover novel function important in the interaction of the bacterium with its host [11]. The rhizobium about which a large amount of proteomics data has been produced is *B. japonicum* cultivated under free or symbiotic conditions [12–15]. Proteomics has also been used to investigate the physiology of other model rhizobia: recently, the proteomes of free living and symbiotic *Mesorhizobium loti* have been described [16], as well as the one of *S. meliloti* [17,18]. None of these studies included bacteroids extracted from stem nodules.

Herein, we determined the proteome of *Bradyrhizobium* sp. ORS278 during root and stem symbiosis with *Aeschynomene indica* in a qualitative approach. In addition, we examine the symbiotic role of some major proteins that emerge from this study and which were not previously identified in a genetic screen [19].

## 2. Results and Discussion

### 2.1. *Bradyrhizobium* sp. ORS278 Samples Overview

In order to obtain new insights into the symbiotic proteomes of *Bradyrhizobium* sp. ORS278, four samples were analyzed, three of which correspond to bacteroids purified from *A. indica* root nodules: two duplicate samples were obtained 14 day after inoculation (day) and one 21 day. Additionally, one sample corresponded to bacteroids purified from stem nodules at 21 day. In total, 1429 bacterial proteins were identified (Table S1) out of the 6749 predicted CDS (21%). The two duplicates sample gave congruent results with respectively 1234 and 1125 proteins identified, 1012 of which being found in the two samples (Table S2). For the 21-day samples, 1146 and 670 bacterial proteins were identified in the root and stem nodules samples respectively. The 14-day and 21-day root nodules proteomes were also very similar with 1076 common proteins. This suggests that bacteroids metabolism does not undergo drastic modification between 14 and 21 days. For the stem nodules, only fewer proteins could be identified, *i.e.*, 670 in total. As compared to root nodules, stem nodules are flatter and likely contain more plant material. This and the difficulty to obtain large amount of stem nodules explain the important differences in protein identifications between stem and root nodules.

### 2.2. Bacteroid Proteomes Are Essentially Encoded by the *Bradyrhizobium* Core Genome

**Figure 1.**
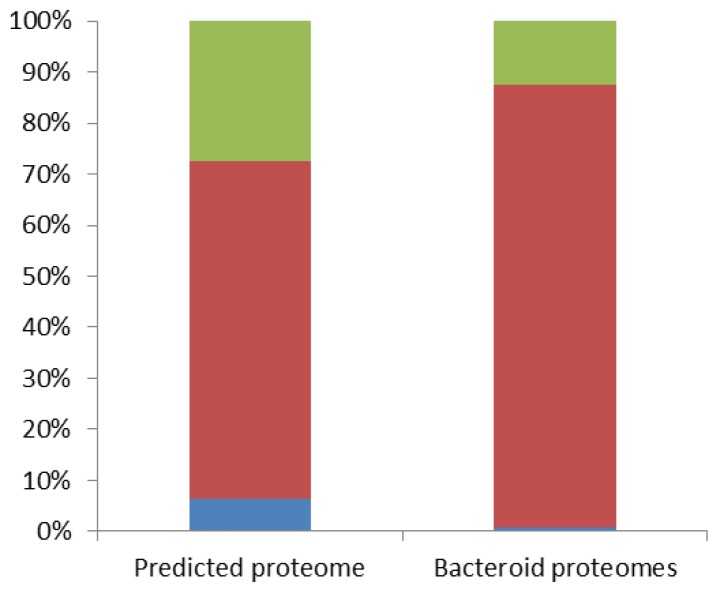
Bacteroid proteomes contain an overrepresentation of core genome encoding proteins and an underrepresentation of ORS278 specific proteins. ORS278 specific proteins are shown in blue, core genome encoding proteins in red and proteins coded by more than one but not all *Bradyrhizobial* genome in green. The distributions of different classes are not similar (homogeneity *CHI*-square test, *DOF* = 2, *p* = 2.2 × 10^−16^).

To analyze the nature of proteins expressed under the different symbiotic conditions, we examined their repartition in the core genome, in the variable genome (*i.e.*, encoded by more than one but not all *Bradyrhizobium* strains) and in the strain ORS278 specific genome. The *Bradyrhizobium* core and variable genomes were determined using the following genomes (ORS278, USDA110, BTAi1, ORS285, ORS375, S23321, STM3809 and STM3843) and the default parameters of gene phyloprofile on the MAGE annotation platform [20] (Table S3). Among the 6749 predicted CDS identified in ORS278 genome, 4460 belong to the core genome (66%), 1865 are variable genes (27.5%) and 435 are specific of the ORS278 strain (6.5%) (Figure 1). In the symbiotic proteomes, we observed an overrepresentation of the core gene encoding proteins that reached 87% and a drastic underrepresentation of proteins encoded by variable and ORS278 specific genes (12% and 1% respectively, Figure 1) suggesting that the metabolism of bacteroids should be well conserved among *Bradyrhizobium* strains in different plant contexts. In addition, these data suggest that the variable genome is more dedicated to free living cells or to the initial steps of the symbiosis rather than alternative bacteroid life style.

### 2.3. Functional Distribution of the Symbiotic Proteins

**Figure 2.**
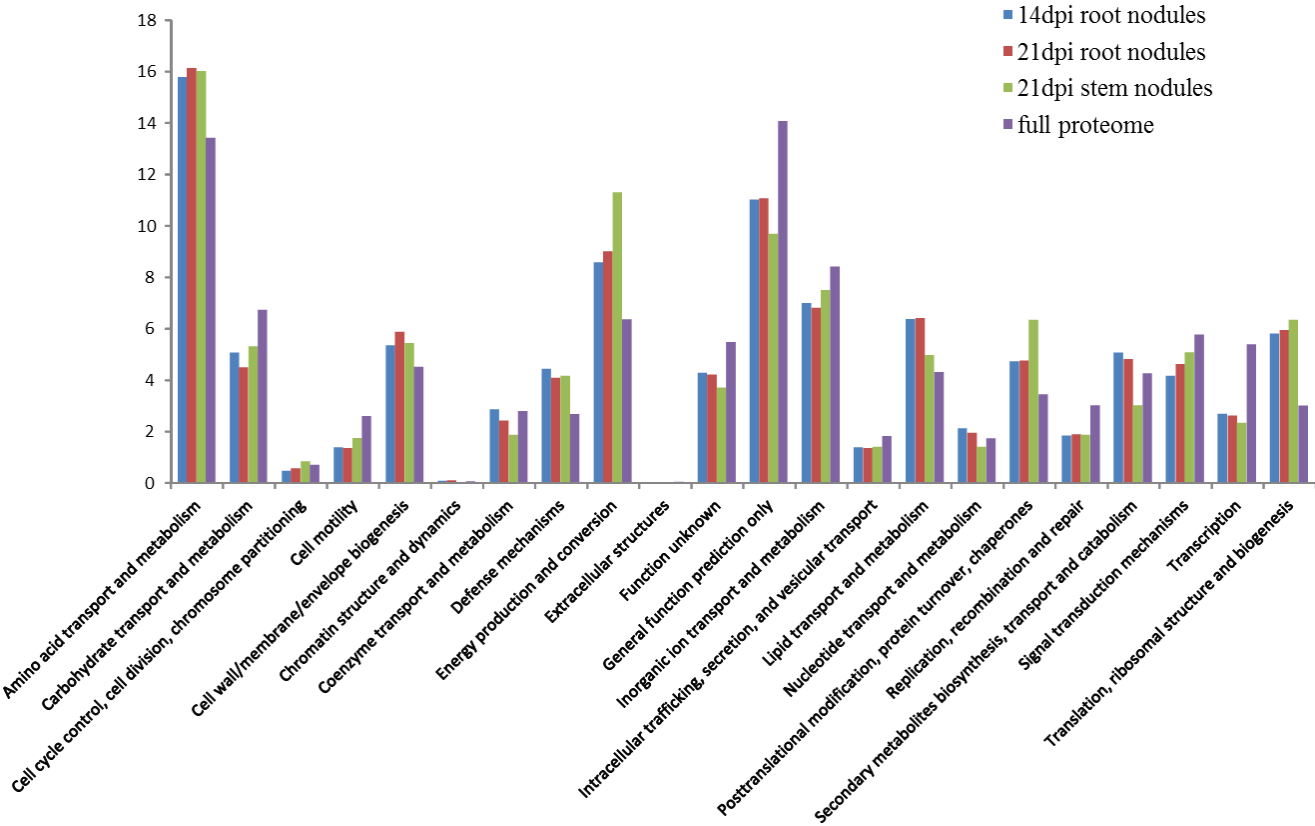
Functional distributions of predicted and bacteroid proteomes according to Clusters of Orthologous Groups (COG) classification, *y* axis represents percentage of the indicated classes.

Functional distribution of proteins of the symbiotic and predicted proteomes were analyzed and compared; the results are illustrated in Figure 2. Amino acid transport and metabolism, defense mechanisms, energy production and conversion, lipid transport and metabolism, post-translational modification, protein turnover, chaperones as well as translational, ribosomal structure and biogenesis are overrepresented in bacteroid proteomes as compared to the full proteome. In contrast, carbohydrate transport and metabolism, cell motility, protein of unknown function or general function prediction only, inorganic ion transport and metabolism, replication, recombination and repair as well as proteins involved in transcription are under-represented in bacteroid proteomes (Figure 2).

### 2.4. Bacteroid Most Abundant Proteins Are Involved in Central Metabolism

**Figure 3.**
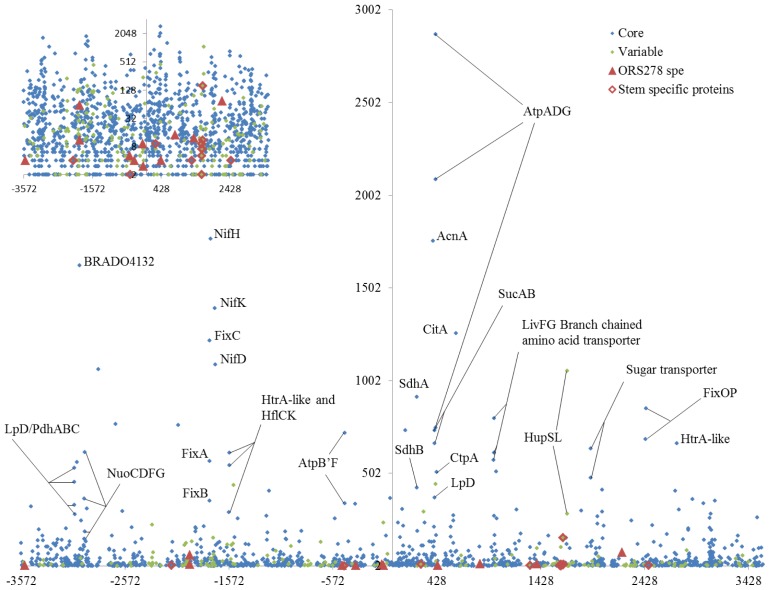
Symbiotic protein distributions, proteins are represented by various markers according to the nature of their corresponding gene and their specificity to the stem bacteroidal proteome. The *y*-axis represents the sum of assigned spectra and *x*-axis the position of the corresponding gene (kbp, position zero being the replication origin). The log scale used in the inset highlights the genome co-localization of most of the stem specific proteins.

Proteins involved in nitrogen fixation (NifHDK, FixABCOP) were amongst the most abundant proteins detected in bacteroids, validating our approach. Proteins involved in ATP synthesis (AtpAB’DGF), microoxic respiration (NuoCDFG), pyruvate metabolism (LpD, PdhABC), and TCA cycle (AcnA, CitA, SdhAB, SucAB, LpD) were also amongst the proteins with the highest number of spectra assigned (Table S1, Figure 3).

Beyond this expected functions, small and large subunits of the hydrogenase uptake (HupS/L) were also highly represented suggesting that nitrogenase evolved hydrogen might be recycled to produce ATP. The abundant presence of hydrogenase in bacteroids is of particular interest since hydrogen production has been described as one of the major factors that affect the efficiency of symbiotic nitrogen fixation [21]. In addition, hydrogenase activity might also result in nitrogenase protection against inhibition by hydrogen, or against excess oxygen [22]. Branched chained amino acid and sugar transporters were also abundant proteins as well as proteases and protease activity modulators (Table S1, Figure 3).

### 2.5. Comparison of Root and Stem Nodule Bacteroids Highlights the Importance of Photosynthesis for Stem Nodulation

Proteomes of stem and root nodule displayed a striking overlay. Amongst the 670 proteins identified in stem nodules bacteroids, only 28 (4%) were not detected in bacteroids isolated from the root nodules at the same age. If we take into consideration the proteomes from bacteroids isolated from 14 dpi root nodules, with all caution due to comparison of nodules collected at different ages, this number drops to 12 (Table S4). Additionally to the bacterial proteins specifically identified in the stem nodules, only two others were found to be induced (spectral count fold change >3 and *p* < 0.05; determined as described in [23]) in stem nodules as compared to root nodules (Table S4). Notably, amongst these 14 proteins, 8 are involved in photosynthesis and are encoded by genes that are co-localized in the same genome region, the photosynthetic gene cluster (Figure 3). The presence of these proteins specifically in the stem nodules sample is an additional argument to validate the proteomic approach. Indeed, it has been previously shown that due to a particular mechanism of regulation by light involving the action of a bacteriophytochrome, the photosystem of ORS278 is formed only in stem nodules [24]. Interestingly, and in agreement with the restricted set of proteins induced in stem bacteroids, only genes involved in photosystems synthesis were found to be regulated by this bacteriophytochrome [25].

### 2.6. Comparison of Proteomics and Genetics Data for Selection of Candidate Proteins for Further Functional Analysis

Recently, 15000 Tn5 mutants were evaluated for their capacity to induce nodules and to fix nitrogen on *A. indica* [19]. As a result, 87 genes were identified as required for efficient symbiotic nitrogen fixation. Out of these, we detected 40 of the corresponding proteins in bacteroids proteomes (Table S5). Reciprocally, amongst the 80 proteins with a number of assigned spectra >100 (in 14 dpi root nodule proteome, Table S6), that can be considered as abundant, 11 were previously described as required for the symbiotic process during the genetic screens (Table S6). This raises the question of the symbiotic role of the other abundant proteins which were not identified during the genetic screen.

Candidate proteins were chosen for further functional analysis based on their annotation and abundance in the nodules. FixA and FixB have been shown to play an essential role in the symbiotic process in other rhizobia [26,27] by supplying electrons to the nitrogenase. Nevertheless, FixA and FixB were not identified during our previous genetic screen. In contrast, mutants altered in *etfA* and *etfB*, that are homologues to *fixA* and *fixB*, respectively were shown to be required for efficient symbiosis in ORS278 [19]. In order to evaluate if EtfA and EtfB fulfill the symbiotic role that FixA and FixB have in other symbiotic systems, BRADO5386 encoding FixA was selected for mutagenesis. Additionally, BRADO0979 encoding a branched chain aminoacid transport protein (LivG), BRADO5083 encoding a conserved hypothetical protein, BRADO1684/1685 encoding the hydrogenase uptake HupS/L, BRADO0393 encoding aconitate hydratase (AcnA), BRADO4479 encoding a putative porin and BRADO4132 encoding an abundant conserved hypothetical protein annotated as containing surface antigen domains were selected for mutagenesis. We used pVO155 [28] to generate insertion mutants and to obtain preliminary results on potential phenotypes. As a consequence, the following results should thus be considered in the light of all drawback of this method such as potential polar effect and reversion to WT genotype after vector excision. Mutants were obtained except for *acnA*, BRADO4132 and BRADO4479, which might reflect an essential role of the abundant selected proteins in bacterial survival.

### 2.7. FixA Is Essential for the Symbiotic Process

**Figure 4.**
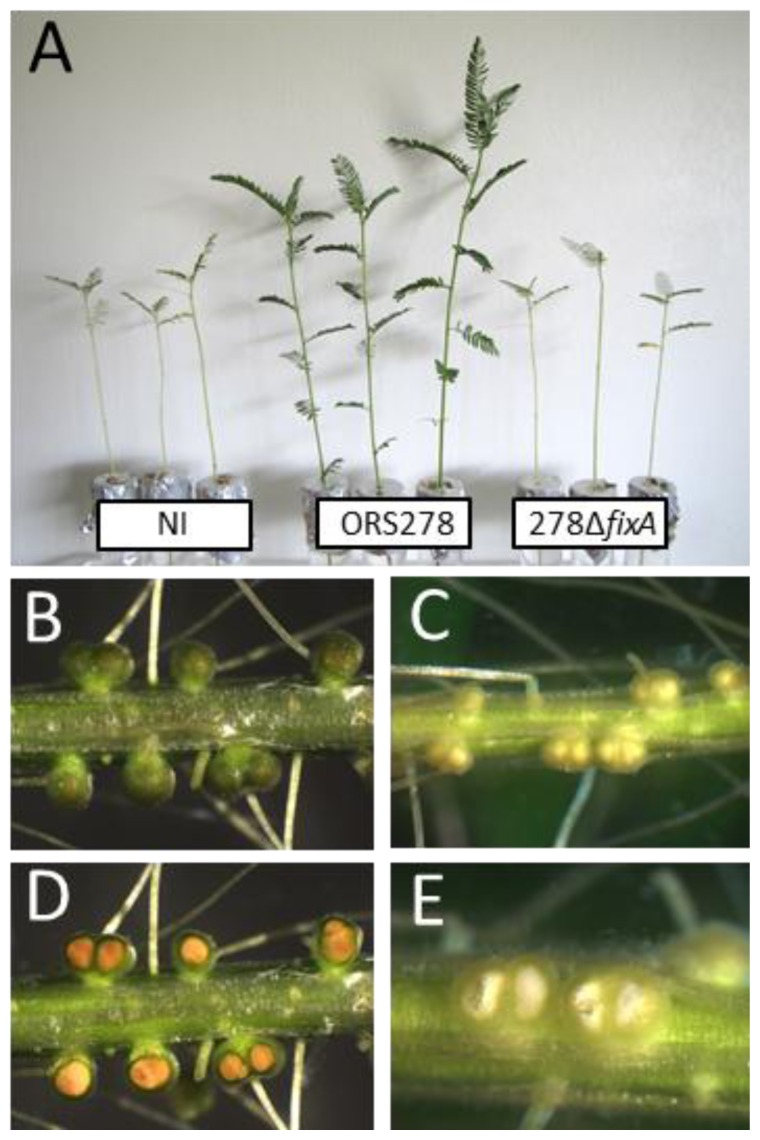
*fixA locus* is required for efficient symbiosis. (**A**) Aerial part of 14 day old plants inoculated with the indicated strain or not inoculated (NI); (**B**) and (**C**) are nodulated root systems of plant inoculated with WT and *fixA::pVO155*, respectively; (**D**) and (**E**) show root systems of plant inoculated with WT and *fixA::pVO155* on which nodules have been sectioned.

Out of the four mutants evaluated only *fixA::pVO155* displayed a symbiotic phenotype. Plants inoculated with this mutant harbored a typical phenotype of nitrogen starvation, *i.e.*, aerial part of infected plants were smaller than those inoculated with ORS278 WT strain and looked similar to those of non-inoculated plants (Figure 4A). Nodules induced by the *fixA::pVO155* mutant displayed the yellowish color of non-functional *Aeschynomene* nodules (Figure 4B,C). Furthermore, observations of *fixA::pVO155* nodule sections clearly indicated that nodules were hollow (Figure 4D,E). Thus, our results suggest that FixAB are also required for the symbiotic process in ORS278, likely by providing electrons to nitrogenase as in other symbiotic systems. EtfA and EtfB proteins were also detected in root nodules, but according to spectral count, these proteins seem far less abundant (10 and 11 spectra for 14 day root nodules samples *vs.* 221 and 148 spectra for FixA and FixB respectively). Their biochemical role in the symbiotic process remains to be elucidated.

## 3. Experimental Section

*Bradyrhizobium* sp. strain ORS278 was cultivated in modified YM medium as described previously [8]. *Aeschynomene indica* seeds were sterilized by incubation in H_2_SO_4_ for 45 min, and washed seven times with distilled sterile water. Seeds were then soaked overnight in water and subsequently transferred onto plates containing 0.8% agar for germination at 30 °C in the dark for 24 h. For 14 day nodule production and for mutant analyses, plantlets were transferred on the top of a test tube covered by a perforated aluminum foil in a way that roots are in contact with liquid buffered nodulation medium (BNM) [29]. For root nodules production, the root system was protected from light by an aluminum foil that covered the test tubes. Roots systems were inoculated as described [30], stems were inoculated using cotton swab soaked in a water washed bacterial suspension OD_600 nm_ = 0.1. All nodules were collected manually and immediately frozen in liquid nitrogen prior to bacteroids isolation.

Bacteroids extraction: five grams for root nodules samples and one for stem were crushed in liquid nitrogen and bacteroids were purified from nodules on a sucrose gradient using a protocol derived from [12] with the modifications described in [13,14].

Proteins were extracted, separated on a mono dimensional SDS PAGE, digested using trypsin and analyzed as described [31]. In Tables S1 and S2 are represented the number spectra assigned for each proteins, in the different samples. For analysis convenience, results from the two 14 day samples were pooled. Fold changes and *p* values are also presented in Tables S1 and S2 for every possible comparison. The used method [23] allowed multiple comparisons with corrections based on the total number of spectra per condition [23].

In order to construct mutant strains altered in *fixA* (BRADO5386), *livG* (BRADO0979), *hupS* (BRADO1684), *acnA* (BRADO0393), BRADO4132, BRADO5083 and BRADO4479, DNA fragments were amplify from ORS278 DNA using the following primer pairs: BRADO5386F (5′-GTGCGTCGA CCTCATCAATCCCTACGACCTGTTC-3′)/BRADO5386F (5′-CCGTCTAGACCTCGTTGATCTTG GCCACATAG-3′); BRADO0979F (5′-CGGTGTCGACGGCGCCGGCAAGACCACACTGTTC-3′)/ BRADO0979R (5′-GGTCTTCTAGACCACCTCCTCGAGATTGAGATAG-3′); BRADO1684F (5′-CTG GCGTCGACGCGCATTGCCAATGCGCTGGAAAC-3′)/BRADO1684R (5′-GCCATCTAGACCTCGG CCATCACCTTCAACTTCTC-3′); BRADO0393F (5′-GAAGGTCGACGGTGACGCCGAGAAGATC AATC-3′)/BRADO0393R (5′-GGCCATCTAGATGACCATGGTGGTGTGCGAATC-3′) BRADO4479F (5′-CTGCTCGTCGACCGGCGCAGGCTTCTACTATATC-3′)/BRADO4479R(5′-GTGAATCTAGAG AACTGGATGAACGCGTTGTAG-3′); BRADO4132F (5′-ATGTCGTCGACGGTCGCGGCTCAGA CGGTCAACTC-3′)/BRADO4132R (5′-CTCGATTCTAGAGGGCGTGACGCGCACGTCATAG-3′); BRADO5083F (5′-CATGCCGTCGACCGGATCGGCGTTCGATTCGTTC-3′)/BRADO5083R (5′-GG CCTCTAGAGGCGAGGCTGGCGAGGAAATAG-3′). Resulting fragments which contained *Sal*I and *Xba*I sites at each extremity were digested and cloned into SalI/XbaI linearised pVO155 [28]. Resulting plasmids were transferred into S17-1 strain to introduce the construction in ORS278 by mating as previously described [32].

## 4. Conclusions

In the present work, we describe the symbiotic proteome of a photosynthetic *Bradyrhizobium* in root and in stem nodules. Furthermore, we show that *fixA* locus is mandatory to establish an efficient symbiosis which is likely involved in providing electrons to nitrogenase as established in other rhizobia and which suggests a so far unknown role for *etfAB* in symbiosis. In addition, our study indicates that the previous mutagenesis studies were not saturating and that some highly abundant proteins are not required to established efficient symbiosis. On the contrary, we did not detect some proteins for which the corresponding genes have been identified previously as required for symbiosis (Table S6). This might be due to low abundance of these proteins, or, to physical properties that are incompatible with proteins extraction or detection. Beyond these technical limitations, the dynamics of the symbiotic process might also be a possible biological reason that might explain the failure to detect proteins essential for symbiosis. Together, our data highlight the complementarity of the genetic and the proteomics approaches.
